# Exploring the associations between the perception of water scarcity and support for alternative potable water sources

**DOI:** 10.1371/journal.pone.0283245

**Published:** 2023-03-17

**Authors:** Christina Semasinghe, Santosh Jatrana, Tanya J. King

**Affiliations:** 1 School of Humanities and Social Sciences, Faculty of Arts and Education, Deakin University, Geelong, Australia; 2 Centre for Rural and Remote Health, James Cook University, Mount Isa, Australia; 3 School of Demography, The Australian National University, Canberra, Australia; 4 Alfred Deakin Institute for Citizenship and Globalisation, Deakin University, Burwood, Australia; University of Ilorin, NIGERIA

## Abstract

This study examines the association between the perception of water scarcity and support for alternative water sources in general, and specifically desalination and recycled water. It also examines the mediating role that perception of climate change has on the aforementioned association. A 46-item survey (n = 588) was conducted in the Geelong region of Australia. Logistic regression was used to determine the independent association between perceived water scarcity and socio-demographic factors, with support for alternative water sources, desalination and recycled water. 82% of respondents supported undefined ‘alternative water sources’. However, support for specific alternatives was lower (desalination: 65%; recycled water: 40.3%). Perception of water scarcity was significantly associated with increased odds of support for alternative water sources (OR 1.94, 95% CI: 1.25–3.00) and support for recycled water (OR 2.32, 95% CI: 1.68–3.31). There was no significant relationship between perception of water scarcity and support for desalination (OR 0.959 95% CI: 0.677–1.358). Climate change was found to mediate perceived water scarcity and support for alternative sources (OR 1.360, 95% CI: 0.841–2.198). The mediation of the relationship between perceived water scarcity and support for recycled water by climate change was not strong. These results facilitate enhanced community engagement strategies.

## Introduction

Water is often taken for granted in regions where it is abundantly found but cherished in regions where the supply is low. Potable water can be attained from a variety of sources. Traditionally these have included rainwater, surface water (e.g. dams and rivers), and groundwater. Globally, traditional sources of fresh water supplies are threatened due to climate change, population growth, and urbanization [1, 2]. Responding to changes in water availability, quality or demand has incorporated an exploration of sources that are ‘alternative’ to the norm. Alternative water sources were defined as tap water sources which don’t rely directly on rainfall, surface or ground water.

Alternative potable water sources can be either dependent on or independent of the climate. Such climate independent sources include wastewater and sea water [3]. In Australia, the driest inhabited continent on Earth, exploring climate independent water sources is a clear necessity. While this requirement may be clear to water agencies, research clearly indicates that public support for alternative water security strategies is vital [4]. Understanding the nuance of community attitudes to various water sources must be a key consideration in future water management planning.

While climate independent water treatment technologies reliably produce high quality potable water [5, 6], public acceptance of these sources presents a major political challenge [7, 8]. Views on alternative potable water sources are varied, and communities are diverse and internally differentiated [9]. Knowledge and attitude are not uniform or stable across different sources and technologies, and are influenced by a dynamic interplay of social, cultural, political, economic, and symbolic factors [10, 11]. While considerable research has been directed towards identifying the socio-demographic characteristics of those more or less likely to accept alternative water sources [11], the results are complex and typically context specific [12]. Broadly speaking, however, wastewater recycling (purified recycled water) typically enjoys less community acceptance, at least in part due to the ‘yuck factor’, the discomfort expressed about the idea of drinking water that is sourced from (particularly toilet) ‘waste’ [13]. Community opposition to desalination tends to focus on environmental and pricing concerns [14]. As Dishman et al. [15 p 154] note, “all of the other problems associated with potable reuse may be resolved, i.e., public health concerns, but the issue of public acceptance remains”.

In the Australian context, two specific infrastructure project events are frequently noted in relation to public support for alternative water sources. The first is the oft-cited 2006 plebiscite that invited residents of the Queensland town of Toowoomba to vote on the introduction of recycled water to their town drinking supply, in the context of an ongoing drought and local dam levels at only eight percent of capacity [16]. Public opposition scuttled the plan despite support from scientists, water agencies and minor celebrities. Concerns about the health, safety and stigma of the plan overpowered those that emphasised the various benefits and safety of the technology. Members of Citizens Against Drinking Sewage (CADS) led nearly two thirds of residents to vote ‘No’ in the referendum [17]. Further, Hurlimann et al. [18] found that residents’ attitudes to Indirect Potable Reuse (IPR) had improved in as little as two years after the ‘No’ vote. Nonetheless, the parable of Toowoomba continues to act as a disincentive to political support for other such recycled water projects around the country [19].

The second event concerns the construction of a desalination plant in the Victorian coastal town of Wonthaggi, which was completed in 2012 [20]. Opposition to the plant was related to the perceived procedural justice of the process of implementation, including inadequate local consultation, the nature of the public private partnerships (PPPs) involved in the plant development, and the compulsory acquisition of private farmland for the plant. Detractors also cited the relatively high costs associated with the plant, as well as a raft of environmental concerns, not least of all being the proximity of the plant to a marine park [8, 21, 22].

It should be noted that both recycled water and desalination plants have been successfully implemented in Australia with far less controversy than in Toowoomba and Wonthaggi (e.g. the rainfall poor state of Western Australia boasts several desalination plants as well as a recently implemented IPR recycled water scheme to supplement the potable supply) [23]. There are positive examples from which water agencies and governments can draw. However, in a risk-averse political climate, negative examples act as a strong deterrent to those seeking to divest from traditional water sources. It is within this broad political narrative context that the current research seeks a clearer understanding of how communities view alternative water sources.

Existing research suggests that perceived need for alternative water sources is linked to concerns about climate change. In the 1970s, Bruvold [24 p.1304] wrote that concerns about drought were a major influence on the attitudes of Californians to water resources and conservation. Similarly, Lam [25 p. 2812] found that when community concern about drought increases, people are more likely to change their behaviours on water conservation. Lam noticed that the community took different water-conservation measures (e.g. modifying their household water appliances) depending on their attitude to water availability. For example, Glick et al. [26 p.5] concluded that Americans who had a strong belief in climate change were more accepting of purified recycled water than those who were less concerned about climate change. While most of the scientific literature support the existence of climate change, the relationship between community perceptions of water scarcity and support for alternative water sources is not clear cut. A person may, for example, be concerned about future access to water but view that as a matter of poor allocation or urban planning. Brownlee et al. [27 p. 964], stress that generic concern about water availability and climate change is not necessarily a strong indicator of water conservation attitudes, unless mediated through concern about location-specific climatic factors (specific instances of drought). In other words, climate change impacts differently from place to place; therefore, these local-level interactions with the climate-impacted resources influence the attitudes to water resources. Evans et al. [28 p. 194] also notes that location specific drought conditions have a significant influence on the community attitude to water scarcity and climate change issues. Community attitudes to the supposed drivers of climate change may affect their attitude to water scarcity [27], and thereby influence their attitudes to the concept of alternative water sources. Even at the level of the individual, a person’s experience in a particular place may either strengthen or weaken their perception of water scarcity and climate vulnerability [29]. However, in contrast to the Brownlee et al. conclusions, Garcia-Cuerva et al. [30] report no significant relationship between experience of severe drought and support for water conservation or the use of recycled water. Likewise, Glick et al. [26] concur that personal experiences of moderate or even extreme drought conditions were not linked to support for recycled water use.

We contribute to this literature by examining the less researched association between perception of water scarcity and support for alternative potable water sources. We initially examine the support for alternative potable water sources without defining what it is meant by the term. The responses given to this initial question were based on the respondents’ pre-conceived understanding of ‘alternative potable water sources’. In a follow-up question we then try to understand their support for specific alternative water sources, namely desalination and recycled water. We examine whether acknowledgment of climate change mediates the relationship between perception of water scarcity and support for alternative water sources. We hypothesize that after adjusting for demographic and socio-economic factors, those who are worried about future drinking water are more likely to support alternative potable water sources.

## Data and methods

### Study area

The research focussed on the Barwon region, in the state of Victoria, south-eastern Australia. Barwon Water (Barwon Region Water Corporation) is the sole water agency that supplies water, sewerage and recycled water services to the region. The region has a population of around 320,00 and is home to the Victorian State’s largest regional city, Geelong. Both the city and the region have undergone considerable expansion and population intensification, increasing 3.3% in 2021 than the previous year [31]. At present, potable water supplies for the region are sourced from catchments on the Barwon and Moorabool rivers, and from an underground aquifer in nearby Anglesea (S1 Fig). There is also a connection to the Victorian water grid via a pipeline from Melbourne to Geelong.

Localised impacts of climate change for two scenarios (i.e. 2030 and 2060) it is predicted that the mean annual rainfall of the Barwon Water catchment region will decline by 2–5% by 2030 and will continue to decline furthermore by 2060 [32]. It is also predicted that the population in the region will be doubled by 2050 [31]. Also, a climatic modelling exercise by Barwon Water has revealed 5 billion litres of water need to be found or saved every five years for the next 50 years. This is in addition to the current demand of 35 billion litres [31].

The topic under research has been of high interest within the water industry in Australia, as diversifying water sources are considered an option to improve water security especially after the prolonged Millennium Drought that lasted from the mid-1990s to 2010 [33]. Water industry has recognised the risk factors of alternative water sources receiving community backlash [34]. On the back of ongoing and anticipated water scarcity, the Victorian water utility, Barwon Water, sought to understand the community’s attitudes to alternative water sources and initiated this research as a collaborative project between Deakin University, Barwon Water and Water Research Australia.

### Survey instrument

A 46-item online survey, taking approximately twenty minutes to complete, was conducted during May–August 2020. The survey questions were created by the authors, based on a thorough literature review [35–38]. The platform Qualtrics was used to develop the survey and contained mostly closed-ended questions. Validity and reliability of our survey instruments were increased by pretesting and piloting the questionnaire. The survey was pre-tested in February 2020 through focus group discussions held in five locations of the region (covering the five local government authority areas). Participant recruitment for these FGDs were done by displaying flyers in public places (supermarkets, libraries etc.) The five FGDs were participated by 12 community members who were asked to answer the questionnaire and give feedback. The average time to complete the survey was recorded. The questionnaire was edited with terminology changed, questions added or deleted based on the feedback given by the participants. The completed piloted questionnaires were closely examined to identify any problems with the questions that might lead to biased questions. A copy of the survey instrument will be included as a supplementary file.

A convenience sample was recruited for the survey by promoting it using a geographically targeted advertisement on social media platforms (Facebook and Instagram). The research was approved by the Deakin University Human Research Ethics Committee (Approval Number HAE 19–249). Written consent of the participants was obtained. The sample was drawn from a total population of 270,000 [39], of those who reside in the region and are above the age of 18. Prior to the survey, the ideal sample size was calculated as 384, using the following formula with a confidence level– 95% and margin of error– 5%) [40]:

Ideal sample size = (Z^2^ * Std Dev* (1-Std Dev))/ (Margin of error)^2^

The survey received a total of 903 responses. However, only 603 survey responses had 100% completion with no missing data. Subsequently, the completed surveys were screened for inclusion and exclusion criteria, which were communicated to the respondents through a plain language statement. Survey responses that were included in the analysis only if the respondents age is above 18 and the postcode was compatible to the region.

The final analysis included 588 survey responses (65.11% of the total responses). The data was analysed using the IBM SPSS statistics software version 27. Respondents were able to further elaborate on some of their responses using qualitative answers. A sensitivity analysis was conducted to determine the impact of removing incomplete and ineligible responses from the final analysis by comparing the demographics and socioeconomic characteristics of the two groups: no statistical difference was identified.

### Variables

#### Perception of future water scarcity

The main exposure used in our analysis was perception of water scarcity which was measured by asking individuals *‘Are you worried about your community’s future access to drinking water*?*’* Responses were recorded on a five-point scale and categorized into two categories that contrasted. If they answered, *‘absolutely yes’* or *‘probably yes’*, the response was categorized as *‘worried’*. If they answered, *‘absolutely not’*, *‘probably not’* or *‘neutral’*, the answer was classified as *‘not worried’*.

#### Socio-demographics

Independent variables controlled for in the analyses included age (18–25 years, 26–60 years, > = 60 years); gender (male, female); level of education (primary/secondary, higher, professional); employment status (employed, not employed/retired/studying/others).

#### Acknowledgment of climate change

The acknowledgment of climate change was measured by asking the individuals *‘Thinking about the last decade*, *have you noticed significant changes in the climate in your region*?*’*. Responses were recorded on a five-point scale. If they answered, *‘definitely have noticed’* or *‘probably have noticed’* it was categorized as *‘yes’* and if they answered, *‘definitely haven’t noticed’*, *‘probably haven’t noticed’* or *‘neutral’*, the answer was classified as *‘no’*.

#### Support for alternative water sources

General support for alternative water sources was measured with the following question: *‘Faced with a drying climate and increasing population our needs and wants may exceed the current supply*. *Do you support the following measures for addressing this shortfall*?*—Add alternative sources of water to the system’*. If the respondent answered yes (either *‘probably yes*’ or *‘absolutely yes*’), their response was coded as *‘yes’* or ‘1’. Negative responses (*‘definitely not*’, *‘probably not*’ or *‘neutral/unsure*’) were coded as *‘no’* or ‘0’.

#### Support for specific alternatives: Desalination and recycled water

A follow up question asked respondents about their support for desalination and recycled water, specifically, using the question: *‘How supportive are you of the following sources of* ***drinking water***?

1. *Desalinated water (sourced from ocean water with the salt removed)*
2. *Purified recycled wastewater (sourced from kitchens*, *laundry*, *toilets*, *and bathrooms)*

Additional options were provided but are outside the scope of this study. Respondents who reported *‘supportive’* or *‘very supportive’* were coded as *‘supportive’* or ‘1’. Negative responses (*‘very opposed*’, *‘opposed*’ or *‘neutral/unsure*’) were coded as *‘not supportive’* or ‘0’.

#### Data analysis

We used both descriptive analysis and binary logistic regression models to investigate the association between perception of water scarcity and support for alternative water sources.

Percentages distribution was used to present respondents characteristics based on sociodemographic and support for alternative potable water sources. Binary logistic regression models were used step by step to examine the association the between perception of water scarcity and support for alternative water sources while controlling for explanatory variables added progressively and cumulatively as follows:

1. Perception of future water scarcity only;
2. Perception of future water scarcity and socio-demographic factors (age, gender, level of education and employment status);
3. Perception of future water scarcity, socio-demographic factors (age, gender, level of education and employment status) and attitudes to climate change.

The findings were presented as odds ratios (unadjusted and adjusted) with a 95% confidence interval. Statistical significance was determined using p<0.05 and p<0.01. The data were analysed using the statistical program Statistical Package for Social Sciences (SPSS), version 27.

## Results

### Sample characteristics

The socio-demographic characteristics of the respondents are given in Table 1. The majority of the respondents were female (58.8%). A higher percentage of respondents were between 26–60 years of age (56%) with the mean age of respondents being 43.4. Around 67% of the respondents had a higher level of education (Bachelor’s degree / graduate diploma /post-graduate degree (e.g. Masters or Doctorate)). Most respondents were employed (56.1%).

**Table 1:** Summary of the socio-demographical data of the respondents.

Characteristics (and categories)	Number	Percent
**Age**		
18–25 years	116	19.6
26–60 years	331	56.3
> = 60 years	141	24
**Gender**		
Female	344	58.5
Male	244	41.5
**Level of education**		
Primary/ Secondary	97	16.5
Tertiary	396	67.3
Professional	95	16.2
**Work status**		
Employed	330	56.1
Not employed/ Retired/ studying/others	258	43.9
**Total**	**588**	**100.0**

The majority (60%) of the respondents said they were worried about the future drinking water supply (Table 2). 66.3% indicated that they had noticed the impacts of climate change in their local environment. The distribution of the responses to water scarcity, alternative water sources and climate change is presented in Table 2. Support for alternative water sources, desalination and recycled water was 83.2%, 65.8% and 40.3% respectively.

**Table 2:** Distribution of the responses to water scarcity, alternative potable water sources and climate change.

Variable	Number	Percent
**Worried about future drinking water**		
No	235	40
Yes	353	60
**Support alternative water sources**		
No	99	16.8
Yes	489	83.2
**Support desalination**		
No	201	34.2
Yes	387	65.8
**Support recycled water**		
No	351	59.7
Yes	237	40.3
**Noticed climate change**		
No	198	33.7
Yes	390	66.3
** Total**	**588**	**100.0**

### Regression results

The regression results show that perception of water scarcity is significantly associated with the support for alternative water sources (OR 1.94, 95% CI: 1.25–3.00) (Table 3, model 1). There was little further change in Model 1 with the addition of socio-demographic factors (OR 1.84, 95% CI: 1.17–2.89) (Table 3, model 2). This suggests that age, gender, employment, and education level were not mediating the association between perception of water scarcity and support for alternative water sources. However, further adjusting for perception of climate change variable (Table 3, Model 3) reduced the perception of water scarcity odds ratio for predicting support for alternative water sources (OR 1.30; 95% CI 0.841, 2.19) making them statistically insignificant, implying that acknowledgment of climate change mediates the association of water scarcity with support for alternative water sources.

**Table 3:** Logistic regression results showing odds ratios (OR) and their 95% confidence intervals (CI) predicting support to alternative potable water sources.

Characteristic	Model 1		Model 2		Model 3	
	OR	95% CI	OR	95% CI	OR	95% CI
**Worried about future drinking water**						
No (R)						
Yes	1.944**	(1.257,3.004)	1.847**	(1.179, 2.892)	1.360	(0.841, 2.198)
**Age**						
18–25 years (R)						
26–60 years			0.774	(0.414, 1.446)	0.814	(0.433, 1.534)
> = 60 years			0.693	(0.335, 1.435)	0.544	(0.258, 1.149)
**Gender**						
Male (R)						
Female			1.102	(0.703, 1.729)	0.948	(0.596,1.509)
**Level of education**						
Primary & secondary (R)						
Higher			1.379	(0.736, 2.584)	1.337	(0.705, 2.535)
Professional			0.749	(0.367, 1.531)	0.745	(0.358, 1.550)
**Work status**						
Employed (R)						
Not employed Retired/studying/Others			1.331	(0.807,2.198)	1.428	(0.883, 2.485)
**Noticed climate change**						
No (R)						
Yes					3.024	(1.852, 4.937)

Perception of water scarcity was not significantly associated support for desalination (OR 0.959; 95% CI 0.677, 1.358) (Table 4, model 1). The socio-demographic variables (OR 0.930; 95% CI 0.649, 1.334) and attitudes to climate change (OR 0.926; 95% CI 0.636, 1.347) does not significantly predict the support for desalination (Table 4, model 2 and model 3). Acknowledgment of climate change does not mediate the association between perception of water scarcity and support for desalination.

**Table 4:** Logistic regression results showing odds ratios (OR) and their 95% confidence intervals (CI) predicting support to desalination.

Characteristic	Model 1		Model 2		Model 3	
	OR	95% CI	OR	95% CI	OR	95% CI
**Worried about future drinking water**						
No (R)						
Yes	0.959**	(0.677, 1.358)	0.930**	(0.649, 1.334)	0.926	(0.636,1.347)
**Age**						
18–25 years (R)						
26–60 years			0.588	(0.358, 0. 965)	0. .589	(0.359, 0. .966)
> = 60 years			1.267	(0.702, 2.287)	1.263	(0.697, 2.288)
**Gender**						
Male (R)						
Female			0.933	(0.653, 1.333)	0.931	(0.649, 1.335)
**Level of education**						
Primary/ Secondary (R)						
Higher			1.150	(0.694,1.907)	1.150	(0.694,1.906)
Professional			1.268	(0.686, 2.344)	1.269	(0.687,2.346)
**Work status**						
Employed (R)			0.898	(0.608, 1.327)	0.900	(0. 609, 1.330)
Not employed/ Retired/studying/Others						
**Noticed climate change**						
No						
Yes					1.019	(0.689, 1.507)

Table 5 presents a significant association between perception of water scarcity and support for recycled water (OR 2.329; 95% CI 1.638, 3.312) (model 1). Age, gender, employment, and education level were not statistically associated with the support for recycled water (OR 2.336; 95% CI 1.618, 3.375) (Table 5, model 2). The association perception of water scarcity and support to recycled water was not substantially changed by the addition of perception of climate change to the regression model 2 (OR 2.048; 95% CI 1.400, 2.997) (Table 5, Model 3) (i.e., the coefficients of perception of water scarcity did not change significantly before and after the inclusion of perception of climate change variable). This suggests that mediation of the relationship between water scarcity and support for recycled water by climate change was not strong.

**Table 5:** Logistic regression results showing odds ratios (OR) and their 95% confidence intervals (CI) predicting support to recycled water.

**Characteristic**	**Model 1**		**Model 2**		**Model 3**	
	OR	95% CI	OR	95% CI	OR	95% CI
**Worried about future drinking water**						
No (R)						
Yes	2.329**	(1.638, 3.312)	2.336**	(1.618, 3.375)	2.048	(1.400,2.997)
**Age**						
18–25 years (R)						
26–60 years			0.839	(0.514, 1.368)	0.855	(0.521, 1.401)
> = 60 years			1.405	(0.807, 2.447)	1.279	(0.728, 2.247)
**Gender**						
Male (R)						
Female			0.581	(0.407, 0.829)	0.542	(0.377,0.778)
**Level of education**						
Primary & Secondary (R)						
Higher			1.878	(1.105, 3.129)	1.871	(1.096, 3.193)
Professional			2.094	(1.120, 3.915)	2.134	(1.135,4.013)
**Work status**						
Employed (R)						
Not employed/Retired/studying/others			1.113	(0.757,1.636)	1.161	(0.787,1.712)
**Noticed climate change**						
No (R)						
Yes					1.710	(1.143, 2.559)

## Discussion

Introduction of alternative potable water sources to the current supply can sometimes be met with public opposition, as with many novel technological concepts. Perception of the intensity and causes of climate change may impact on how one perceives water scarcity, and this can impact community support for alternative water sources. This study examined the association between the perception of water scarcity and support for alternative potable water sources focussing on general attitudes towards alternative supplies, and specifically desalination and recycled water.

The present study has three major findings:

1. Perception of water scarcity was a significant predictor of support to alternative water sources (Table 3, Model 1) and recycled water (Table 5, Model 1).
2. Perception to climate change mediated the relationship between perception of water scarcity and support to alternative water (Table 3, Model 3), however, it did not mediate the relationship between perception of water scarcity and support to recycled water (Table 5, Model 3).
3. Perception of water scarcity did not predict support to desalinization (Table 4, Model 1)

82% of the respondents recorded support alternative water sources when not specified. This suggests that with a high awareness of climate change among the respondents (66.3%), there is an understanding that solutions should be sought to replenish declining water resources. However, a follow-up question inquiring about support for desalination and recycled water received 65.8% and 40.3% support respectively. The explanation to this result could be that the term ‘alternative’ has a positive connotation and therefore receives higher support from respondents. The terminology used in the question also seems to play a crucial role in shaping the responses. The term ‘alternative’ implies that a new source outside the established water supply sources. But, when specific types of alternative potable water sources were defined (e.g. desalination, recycled wastewater) the support of the respondents declined.

There could be several explanations for why the perception of water scarcity was associated with support for alternative potable water sources and recycled water. It may be that due to the frequency of droughts in Australia that the community recognizes the need for an alternative water supply plan. Those who are worried about their future access to drinking water are significantly more likely to express support for accessing water from non-traditional sources. This finding is consistent with research done by Hurlimann & Dolnicar [19] who found that the majority of participants agreed they would support recycled water if a drought situation worsened (p. 290). Support for alternative water sources is higher in areas where water is scarce [41]. However, Garcia-Cuerva et al. [30] found that the experience of water scarcity is not a clear predictor of support for water recycling in a study done in America, though it has been found to increase self-assessed water conservation behaviors and general concerns about water scarcity.

The present study also found that the association between perceptions of water scarcity and support for alternative potable water sources was mediated by attitude to climate change (Table 3 Model 3). When attitude to climate change was controlled in Model 3 of Table 3, the relationship between perceptions of water scarcity and support for alternative potable water sources became non-significant. That suggests that for perception of water scarcity to promote the support for alternative potable water sources, the perception of climate change is a vital factor. In other words, without recognizing climate change as a threat to water security, support for alternative potable water sources may not be achieved. Thus, perception of water scarcity does not directly lead to support for alternative potable water sources. Rather, a perception of climate change, combined with perception of water scarcity, is a significant influential factor that leads to support for alternative potable water sources.

However, in contrast, the results showed that perception of climate change did not mediate the relationship between perceptions of water scarcity and support for recycled water. There was minimal change in perceptions of water scarcity coefficients with the introduction of perception of climate change, indicating an independent effect of perceptions of water scarcity on support for recycled water. It can be assumed that the association between perceptions of water scarcity and support for recycled water could be mediated through other factors such as population growth, inappropriate distribution, or changes in rainfall distribution that occur due to natural variation (not climate change), which need to be explored in future studies.

Contrary to the support for alternative potable water sources and recycled water, our results showed that there was no significant relationship between perception of water scarcity and support for desalination (Table 4, Model 1). The Australian ‘parables’ of Wonthaggi (desalination) and Toowoomba (recycled water) should be taken into consideration when considering public attitudes. Indeed, qualitative research associated with this project suggest that these stories contribute to the framing of discussions about alternative water supplies.

The concern of the public about future water supplies suggests that there is general overall support for alternatives sources of water to be added to the supply in the Barwon region. However, water agencies and governments should be mindful of the context in which the precise sources are canvassed, discussed, and introduced, with particular attention to the context in which the discussion emerges.

## Strengths and limitations of the study

This study had several limitations. First, this study reports cross-sectional analyses, which prohibit drawing causal conclusions. Second, we used only a single measurement of perception of water scarcity. We were not able to measure attitudes to water scarcity due to population growth, urbanisation, and other disparities in water distribution systems. We were also not able to check the association of the yuck factor and support to different alternative potable water sources. It would be of interest for future research to examine how the yuck factor mediates the support to alternative potable water sources. This would especially be important in relation to support to recycled water, as literature points out to the opposition to recycled water due to the negative connotation it has with the association it has with wastewater. Further research should also consider institutional and political factors such as trust and procedural justice, which are considered as key factors that determine the level of support to alternative potable water sources.

## Conclusion

Ensuring the availability of adequate water for a growing population on the driest inhabited continent on Earth is a key challenge, particularly in the context of climate change. This is a challenge faced not only by water agencies but by politicians who must convince the public to accept alternative supplies. While the water technology is thoroughly capable of treating alternative water sources to potable drinking standards [3, 42], public acceptance is a key factor in the successful implementation of an alternative water schemes [19]. Understanding the relationships between public attitudes to supply and acceptance of alternatives plays an integral part in the overall goal of securing adequate supplies of water that are both scientifically and politically appetizing.

The results of this paper find that a higher awareness of water scarcity increases the acceptance of alternative potable water sources, specifically for recycled water, but not for desalination. Socio-demographic data did not mediate the association between the perception of water scarcity and support for alternative potable water sources. Perception of water scarcity was not significantly associated with support for desalination. However, acknowledgment of climate change mediates the association of water scarcity with support for alternative potable water sources. But mediation of the relationship between water scarcity and support for recycled water by climate change was not significant.

From an applied point of view, the perception of water scarcity should be considered when designing longitudinal surveys to assess community attitudes to alternative potable water sources. The findings also provide other potentially useful insights for climate change outreach programs. The insights gained through the study will have implications when designing interventions to influence community attitudes to alternative potable water sources.

## Supporting information

S1 Fig Map of study region. (TIF)

S1 File Survey instrument. (PDF)

S1 Data (XLSX)
