# Supplementary figures and images for: Exploring the associations between the perception of water scarcity and support for alternative potable water sources
Source: PLoS One. 2023 Mar 17;18(3):e0283245. doi: 10.1371/journal.pone.0283245 (PMC10022764; doi:10.1371/journal.pone.0283245)

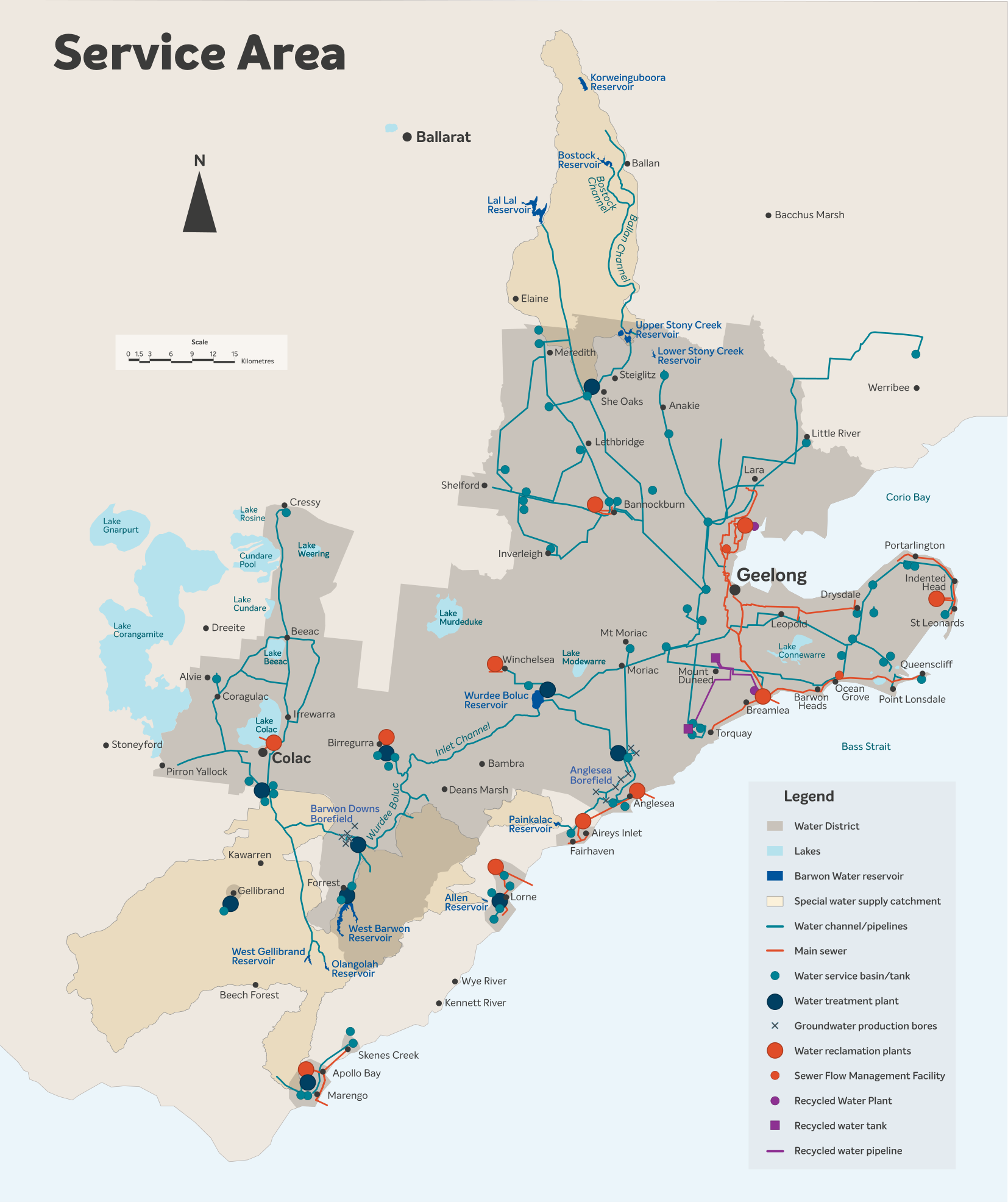

Supplement: S1 Fig — (TIF) [file pone.0283245.s001.tif]
