# Supplementary material for: Exploring the associations between the perception of water scarcity and support for alternative potable water sources
Source: PLoS One. 2023 Mar 17;18(3):e0283245. doi: 10.1371/journal.pone.0283245 (PMC10022764; doi:10.1371/journal.pone.0283245)
Supplement: S1 File — (PDF) [file pone.0283245.s002.pdf]

The online survey

You are invited to take part in this survey about your attitudes to alternative sources of drinking water. This research is done as a part of the PhD study of Ms. Christina Semasingha and is supervised by Dr. Tanya King of the School of Humanities and Social Science, Faculty of Arts and Education, Deakin University, Waurn Ponds campus, Geelong. The survey should take around 20-25 minutes to complete. The survey period is from Monday 27th April to Sunday 14 July 2020. As a thank you for participating, you may enter into a rolling weekly prize draw for one of forty $50 gift cards (ten per month for the four months of the survey), or a $50 donation to a registered charity of your choice.

Please read the Plain Language Statement before you start the survey. Thank you for your valuable time and participation in this survey.

Start of Block: What do you think about your current water supply?

These questions tell us about your current water use, experiences and behaviours. Don't spend too long thinking about the answers, but rather go with your initial thoughts. There are no right or wrong answers!

1. For water that you drink do you usually:
   - ☐ Use tap water, boiling it first
   - ☐ Drink it straight from the tap
   - ☐ Use tap water filtered (e.g. on your tap, or via a standalone filtration system)
   - ☐ Buy bottled water
   - ☐ Collect rainwater
   - ☐ Other- please list ____
2. Which of the following best represents the **primary** water supply to your home?
   - ☐ Mains water (supplied by my water utility to my property)
   - ☐ Bore water on my property
   - ☐ Rainwater tanks on my property
   - ☐ I don't know - but I think I should know more
   - ☐ I don't know - and I don't really care
   - ☐ Other- please list ____
3. Do you know who supplies water to your home?
   - ☐ City Council
   - ☐ Barwon Water
   - ☐ Private industry
   - ☐ Victorian Water
   - ☐ Other ____
4. Water piped to homes by Barwon Water comes from a treatment plant, where it is purified and made safe to drink. Which of the following sources **do you think** supply the treatment plant? (Multiple answers possible)
   - ☐ Surface water (dams, rivers, reservoirs)
   - ☐ Rainwater tanks at the treatment plant
   - ☐ Groundwater (water in underground lakes and aquifers)
   - ☐ Wastewater (toilets)
   - ☐ Wastewater (kitchen, bathrooms and laundry)

- ☐ Urban storm water
- ☐ Desalination plant (ocean water with the salt removed)
- ☐ I don't know - but I think I should know more
- ☐ I don't know - and I don't really care
- ☐ Other ____

5. In general, how would you describe your satisfaction with the quality of your piped household water supply?

Smell	Very unsatisfied	Unsatisfied	Neutral	Satisfied	Very satisfied
Taste	Very unsatisfied	Unsatisfied	Neutral	Satisfied	Very satisfied
Appearance	Very unsatisfied	Unsatisfied	Neutral	Satisfied	Very satisfied

6. Overall, rate your satisfaction of your household water supply?
   - ☐ Very unsatisfied
   - ☐ Unsatisfied
   - ☐ Neutral
   - ☐ Satisfied
   - ☐ Very satisfied
7. Do you currently reuse water from your home?
   - ☐ No
   - ☐ Maybe
   - ☐ Yes
8. What do you reuse water for in your home? (Multiple responses possible)
   - ☐ Toilet flushing
   - ☐ Garden watering-lawn
   - ☐ Garden watering-vegetables
   - ☐ Car washing
   - ☐ Clothes washing
   - ☐ Washdown of external surfaces
   - ☐ Any other uses- please list (separated by commas) ____
9. What are the reasons you don't reuse water in your home?
   - ☐ I am too busy
   - ☐ Inbuilt systems are too expensive
   - ☐ I don't think there's an environmental need to reuse water in my home
   - ☐ I don't really think about it
   - ☐ It might make me sick
   - ☐ It seems disgusting
   - ☐ It’s not offered to me by my water utility
   - ☐ I'm in a rented property
   - ☐ Other ____
10. Do you consider yourself to be an environmentally minded person?

- ☐ No
- ☐ Maybe
- ☐ Yes

11. Do you do any of the following? If so, why? (Multiple responses possible)

	Environmental reasons	To save money	Social reasons	Other reasons
Minimise packaged purchases				
Buy/sell/swap/donate secondhand goods				
Use rainwater collected and stored in tanks				
Manually divert wastewater (e.g. using buckets or hoses)				
Try to use less water (e.g. short showers, turn tap off while brushing teeth)				
Use water-saving devices (e.g. low-flow showerheads)				
Grow own food				
Limit meat consumption				
Use a composting toilet				

Start of Block: What do you think about the amount of water available for your region?

In this set of questions, we ask what you think about how much water is available for your region. It doesn't matter if you don't know too much about this topic - we are interested in what you think.

12. Thinking about the last decade, have you noticed significant changes in the climate in your region?

- ☐ Definitely haven't noticed
- ☐ Probably haven't noticed
- ☐ Might or might not have noticed
- ☐ Probably have noticed
- ☐ Definitely have noticed
- ☐ UNSURE

13. Thinking about the last decade, have you noticed changes to any of the following in your region?

	Much lower	Lower	No change	Higher	Much higher	UNSURE

Rainfall
Temperatures
'Unusual' weather
Bushfire
Flooding
Droughts

14. What do you think is the main driver of climate change **globally**? (Multiple answers possible)

- ☐ Divine influence (e.g. an act of God or gods)
- ☐ Natural climatic variation
- ☐ Human activities, such as driving cars and burning fossil fuels
- ☐ Increasing population
- ☐ Other ____
- ☐ I don't believe there has been significant change

15. Are you worried about your community's future access to water?

	Absolutely Not	Probably Not	Neutral	Probably Yes	Absolutely Yes	UNSURE
Drinking water						
Water for inside use (e.g. showering)						
Water for outside use (e.g. garden)						
Water for municipal use (e.g. swimming pools, watering sports ovals)						
Water for agricultural use (e.g. irrigation)						
Water for industrial use (e.g. manufacturing)						
Other						

16. Our community needs water. Faced with a drying climate and increasing population our needs and wants may exceed the current supply. Do you support the following measures for addressing this shortfall?

	Definitely not	Probably not	Neutral	Probably yes	Definitely yes	UNSURE
Add alternative sources of water to the system						
Water restrictions						

Education campaigns to reduce water consumption	
Address climate change	
Regulate regional population density	
Reduce industrial use	
Reduce agricultural use	
Reduce meat production/consumption	
Improve pipelines to reduce leaks and wastage	
Invest in water-saving technology (e.g. household digital meters for leak detection)	
Other	

Start of Block: What do you think about sources of drinking water?

This section asks you about your attitudes to different sources of drinking water. We're interested in both your level of support for various sources, and which source you prefer.

17. How supportive are you of the following sources of **drinking water**? This question is intentionally very general; you'll have an opportunity to explain your answers, below.

	Very opposed	Opposed	Neutral	Supportive	Very supportive	UNSURE
Rainwater tanks						
Dams or reservoirs						
Groundwater or aquifers						
Desalinated water (ocean water with the salt removed)						
Urban stormwater						
Purified recycled wastewater (from kitchens and laundry)						
Purified recycled wastewater (from						

toilets and bathrooms)	
Other	

18. Drag and drop to rank the following sources of tap water in order of your preference, with your most preferred option (1) and your least preferred option (8).

- Rainwater tanks
- Dams or reservoirs
- Groundwater or aquifers
- Purified recycled wastewater (from kitchens and laundry)
- Purified recycled wastewater (from toilets and bathrooms)
- Desalinated water
- Urban stormwater
- Other

The following questions are on alternative tap water sources which don't rely directly on rainfall (climate-independent alternative water sources). In this research we are focusing on desalinated water, and different sources of recycled water, because these are the most commonly used climate-independent alternative water sources globally.

In all cases, please assume that the water is treated to a very high standard and meets all Government and regulatory safety criteria (it's safe to drink).

19. Do you have concerns about: **Desalination** (sourced from ocean with the salt and other impurities removed)?

	Definitely not	Probably not	Neutral	Probably yes	Definitely yes	UNSURE
Safety for human consumption						
Safety for industrial use						
Environmental impact						
Cost of infrastructure (i.e., capital investment)						
Cost to the consumer (i.e. your household bill)						
Transparency of decision-making process						
Other						

20. Do you have concerns about: **Purified recycled wastewater** (from kitchens and laundry)?

	Definitely not	Probably not	Neutral	Probably yes	Definitely yes	UNSURE

Safety for human consumption						
Safety for industrial use						
Environmental impact						
Cost of infrastructure (i.e., capital investment)						
Cost to the consumer (i.e., your household bill)						
Transparency of decision-making process						
Other						

21. Do you have concerns about: **Purified recycled wastewater** (from toilets and bathrooms)?

	Definitely not	Probably not	Might or might not	Probably yes	Definitely yes	UNSURE
Safety for human consumption						
Safety for industrial use						
Environmental impact						
Cost of infrastructure (i.e. capital investment)						
Cost to the consumer (i.e. your household bill)						
Transparency of decision-making process						
Other						

22. Would you be MORE likely to support **purified recycled wastewater** (from toilets and bathrooms) if it were stored in **an environmental buffer** (e.g. a dam or in groundwater) for a period of time before reincorporation into the drinking water system?

- ☐ Yes, definitely
- ☐ Yes, but it would depend on HOW LONG it was stored (how long?) ____
- ☐ Yes, but it would depend on WHERE it was stored (e.g., dam, aquifer) ____
- ☐ Yes, but it would depend on the SYSTEM SET-UP COST
- ☐ Yes, but it would depend on the cost to my HOUSEHOLD BILL
- ☐ Actually, I would SUPPORT THE DIRECT REINCORPORATION (without an environmental buffer) of treated recycled toilet water into the drinking system, as long as it was safe to drink
- ☐ No, I would NEVER support the reincorporation of recycled water into the drinking system
- ☐ Other ____

23. This question asks about the 'yuck factor', a concept known in the water industry for the 'instantaneous reactions/ first instinct' people have to different kinds of water sources. This response is not necessarily shaped by technical knowledge or scientific evidence but is a more emotional reaction and may be influenced by your background, culture, or other beliefs. There is no right or wrong answer to this question, so please answer as honestly as you can.
What are your 'instantaneous reactions' to drinking water from the following sources (you can choose multiple emotions):

	Unsure	Satisfied	Yuck!	Angry	Upset	Don't care as long as it is safe
Desalinated water						
Purified recycled wastewater from bathrooms, kitchen and laundry (directly reincorporated)						
Purified recycled wastewater from toilets (directly reincorporated)						
Purified recycled wastewater from bathrooms, kitchen and laundry (reincorporated after storage in environmental buffer)						
Purified recycled wastewater from toilets (reincorporated after storage in environmental buffer)						

Start of Block: How do you feel about your water utility?

In this section we ask you some questions about your attitude to your water utility, the organisation that supplies your home with water. Some of these questions may sound repetitive, but please answer them all, as these are carefully designed to better enable us to determine community attitudes.
We want to stress that no water utility (not Barwon Water or any other water utility) will have access to individual survey responses, so please answer as honestly as possible.

24. Below are some questions about how you view your water utility.

	Strongly disagree	Disagree	Neutral	Agree	Strongly agree	UNSURE
Overall, I'm treated fairly by my water utility						

Usually, the way things work in my local water utility are fair
In general, I can count on my water utility to be fair
In general, the treatment the community receives from my water utility is fair

25. How much do you trust your main water utility to provide you with safe drinking water?

- ☐ Highly distrust
- ☐ Moderately distrust
- ☐ Neutral
- ☐ Moderately trust
- ☐ Highly trust
- ☐ UNSURE

26. Have you ever lodged a complaint to your water utility?

- ☐ No
- ☐ Yes

27. If yes, what was it about? (Multiple choices possible)

- ☐ Water supply pressure
- ☐ Water quality-colour
- ☐ Water quality-odour
- ☐ Water quality-taste
- ☐ Billing
- ☐ Customer support
- ☐ Water extraction
- ☐ Other

28. Were you satisfied with the way your water utility resolved your problem?

- ☐ No
- ☐ Yes

29. Would you like to explain your answer?

____

30. How would/do you prefer to get information from your water utility? (Multiple answers are possible)

- ☐ Water utility information stall (e.g. at shows, fairs or other events, or as stand-alone stalls)
- ☐ Water utility public workshop or seminar (e.g. focussed event at a specific time/place)
- ☐ Water utility website
- ☐ Water utility brochure or leaflet delivered in the post
- ☐ Print media, radio and/or television
- ☐ In-school programs

- ☐ Social media
- ☐ Other ____

31. What sources of information would you find persuasive when deciding whether or not to support climate-independent alternative water sources, such as desalination and recycled water?

	Not important	Somewhat important	Very important	UNSURE
Scientific reports				
Economic reports				
Victoria Government reports				
International case studies (e.g. Singapore)				
National case studies (e.g. Perth)				
Endorsement by politicians				
Endorsement by friends and family				
Endorsement by celebrities (e.g. Geelong Football Club)				
The opportunity to try the water				
Media reports				

32. If your community overwhelmingly voted to incorporate alternative sources of water into the regular supply (i.e. to homes), and it was treated so that it was safe to drink, would you:

- ☐ Drink it straight from the tap
- ☐ Use tap water filtered (e.g., on your tap, or via a standalone filtration system)
- ☐ Use tap water, boiling it first
- ☐ Buy bottled water
- ☐ Other ____

Start of Block: Tell us a little about yourself

These questions help us to understand who has responded to our survey, and how closely the responses match the composition of the region as a whole. These questions also allow us to see how different sectors of the community (e.g., women and men, rural and urban people), view alternative sources of drinking water. They might seem simple, but these questions are a very important part of any survey.

33. What is your gender?

- ☐ Male
- ☐ Female
- ☐ Other (specify) ____
- ☐ Prefer not to say

34. How old are you?

35. Were you born in Australia?

36. If no -In which country, were you born?

37. In which year did you first arrive in Australia?

38. Are you of Aboriginal or Torres Strait Island Descent?

- ☐ No
- ☐ Yes- Aboriginal
- ☐ Yes - Torres Strait Islander
- ☐ Yes- Aboriginal and Torres Strait Islander
- ☐ Decline to state

39. Did you grow up mostly:

- ☐ In a capital city
- ☐ In a regional city
- ☐ In a country town
- ☐ On a farm
- ☐ On a hobby farm or peri urban residence
- ☐ Other (Specify) ____

40. What is the postcode of your main residence?

41. Who do you live with?

- ☐ No one- I live alone
- ☐ Spouse/partner
- ☐ Spouse/partner and children (either above or under 18)
- ☐ With children (either above or under 18)
- ☐ Share house (non-relatives)
- ☐ Aged care center/ Retirement village
- ☐ Pet/s
- ☐ Other ____

42. How old are the children living in your home? (Separate ages with a comma)

43. What is the highest level of education you have completed?

- ☐ Did not have much formal schooling
- ☐ Primary School
- ☐ Junior Secondary / Intermediate / Form 4 / Year 10
- ☐ Senior Secondary / Leaving / Form 6 / Year 12
- ☐ Certificate (Level I, II, III or IV)
- ☐ Trade Certificate
- ☐ Diploma or Advanced Diploma
- ☐ Bachelor’s degree
- ☐ Graduate Certificate or Graduate Diploma
- ☐ Post-graduate Degree (e.g., Masters or Doctorate)
- ☐ Other ____

44. What is your usual employment status (prior to any changes driven by the Corona virus COVID-19)?

- ☐ Working full-time
- ☐ Working part-time
- ☐ Not employed, but looking for work
- ☐ Pension
- ☐ Retired
- ☐ Studying full-time
- ☐ Studying and working part-time
- ☐ Prefer not to say
- ☐ Other (Specify) ____

45. Has your employment status been impacted by the Corona virus, COVID-19? Please explain.

- ☐ No
- ☐ Yes (Please explain) ____

46. What is your usual total household income, before taxes last financial year? (AUD, 000)

- ☐ 25
- ☐ 50
- ☐ 75
- ☐ 100
- ☐ 125
- ☐ 150
- ☐ 175+
- ☐ Prefer not to say

47. Do you consider yourself to be an active user of social media?

- ☐ No
- ☐ Yes

48. What social media platforms do you use? (Multiple answers are possible)

- ☐ Facebook
- ☐ Twitter
- ☐ Instagram
- ☐ YouTube
- ☐ LinkedIn
- ☐ WhatsApp
- ☐ Houseparty
- ☐ WeChat
- ☐ TikTok
- ☐ Google+
- ☐ Other ____

49. What are your sources of news? (Multiple answers are possible)

- ☐ Radio
- ☐ Television
- ☐ Online

☐ Friends and family

☐ Print media (e.g., newspapers)

☐ Social Media

☐ Other ____

Start of Block: Invitation to a focus group discussion

We wish to hear more about your thoughts on alternative water sources and would like to invite you to participate in a Focus Group Discussion.
We will ask you to enter your contact information, which will kept separate to your survey answers.

Do you wish to take part in a **Focus Group Discussion** and/or **Enter the Prize Draw**

☐ Yes

☐ No
